# Evaluating Costs and Efficacy of rFVIII Prophylaxis Using Matching-Adjusted Indirect Comparisons in Hemophilia A

**DOI:** 10.1055/a-2923-0928

**Published:** 2026-08-24

**Authors:** Craig M. Kessler, Pier M. Mannucci, Victor Jiménez-Yuste, Laurent Frenzel, Benoit Guillet, Fernando F. Corrales-Medina

**Affiliations:** 1 Lombardi Cancer Center, Medstar Georgetown University Medical CenterWashingtonDistrict of ColumbiaUnited States; 2 Department of Scientific DirectionIRCCS Ca' Granda Maggiore Policlinico Hospital FoundationMilanItaly; 3 Hematology and Hemotherapy Unit, La Paz University Hospital (IdiPAZ)MadridSpain; 4 16722 Faculty of Medicine, Universidad Autonoma de MadridMadridSpain; 5 246596 Department of Haematology, Hôpital Necker-Enfants maladesParisÎle-de-FranceFrance; 6 36684 Haemophilia Treatment Center, University Hospital Centre RennesRennesBretagneFrance; 7 27079 Inserm, EHESP, Irset (Institut de recherche en santé, environnement et travail - UMR_S 1085), Université de RennesRennesBretagneFrance; 8 12235 Miller School of Medicine, University of MiamiMiamiFloridaUnited States; 9 University of Miami – Hemophilia Treatment CenterMiamiFloridaUnited States

## Abstract

**Introduction** Prophylaxis with factor VIII (FVIII) is the standard of care for individuals with severe hemophilia A, but it is associated with a substantial economic burden. This analysis compared the efficacy, dosing, and annual treatment costs of simoctocog alfa (Nuwiq, a recombinant FVIII [rFVIII]) with five extended half-life (EHL) rFVIII concentrate products.

**Methods** Matching-adjusted indirect comparisons (MAICs) were performed to compare simoctocog alfa with efanesoctocog alfa (ALTUVIIIO) and turoctocog alfa pegol (Esperoct), and results were integrated with a previously published MAIC comparing simoctocog alfa with efmoroctocog alfa (ELOCTATE), damoctocog alfa pegol (JIVI), and rurioctocog alfa pegol (ADYNOVATE). These MAIC-adjusted populations were used to develop a cost model from a United States payer perspective.

**Results** Annual total costs per person were $147,058 to $492,090 lower with simoctocog alfa compared with EHL rFVIII products, corresponding to a 21% to 46% cost reduction, primarily driven by lower drug acquisition costs. Outcomes were comparable or favorable to simoctocog alfa versus turoctocog alfa pegol, efmoroctocog alfa, damoctocog alfa pegol, and rurioctocog alfa pegol. In comparison with efanesoctocog alfa (Group A), no significant differences were observed in the proportion of individuals with zero bleeds, whereas treated total annualized bleeding rate (ABR) and treated spontaneous ABR favored efanesoctocog alfa.

**Conclusion** This indirect comparison suggests that personalized prophylaxis with simoctocog alfa may offer economic advantages versus EHL rFVIII products in individuals with severe hemophilia A. Clinical outcomes were broadly comparable across comparators, although treated total and spontaneous ABRs were significantly lower with efanesoctocog alfa (Group A).

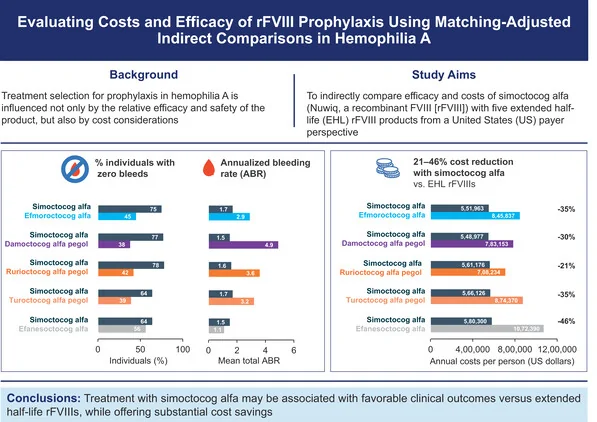

## Introduction

Severe hemophilia A is associated with a significant health and economic burden. 1 2 3 Prophylaxis is the standard of care to prevent recurrent bleeding in individuals with severe disease. 1 Prophylaxis with factor VIII (FVIII) replacement therapy should be personalized based on bleeding phenotypes, pharmacokinetics (PK), joint status, and lifestyle factors. 1 4

Recombinant FVIII (rFVIII) concentrates are categorized as standard half-life (SHL) or extended half-life (EHL) products. EHL rFVIIIs incorporate structural and chemical modifications to prolong circulation time. 1 5 6 Treatment selection is influenced not only by the relative efficacy and safety of the product, but also by cost considerations. The economic impact is considerable in the United States, with the average annual direct costs of FVIII prophylaxis reported to be $656,707, with FVIII replacement therapy accounting for 97% of costs. 3

Simoctocog alfa (Nuwiq, Octapharma AG) is a B-domain-deleted rFVIII produced in a human cell line, without protein fusion or chemical modifications, approved for bleeding prevention and treatment in individuals of all ages with severe hemophilia A. 7 8 9 10 11 12 13 The phase IIIb NuPreviq study demonstrated the efficacy and safety of PK-guided, personalized prophylaxis with simoctocog alfa in previously treated persons (PTPs), with over 80% of participants experiencing zero spontaneous bleeds over a 6-month period. 12 Notably, more than half of participants received dosing twice weekly or less, with a reduced mean weekly effective FVIII dose compared with prior standard prophylaxis with simoctocog alfa. 12

Comparative cost analysis of rFVIII products must consider dosing strategies to achieve adequate bleed protection, the half-life of FVIII replacement products, breakthrough bleeding rates, and the cost of factor replacement in the prevention and treatment of bleeds. However, direct comparisons from distinct peer-reviewed published clinical trials are limited by small sample sizes and heterogeneous trial designs. 14 In the absence of head-to-head trials, matching-adjusted indirect comparison (MAIC) offers a validated approach to minimize bias by adjusting individual participant-level data (IPD) from an index study to match the baseline characteristics of aggregate comparator populations from a second study. 15 16 17 18 19 20 MAIC is being increasingly used across therapeutic areas, 19 including hemophilia A. 21 22 23 24 25 26 27 These analyses can also support economic modeling by providing comparative costs of treatments.

The aim of this study was to compare the total annual treatment costs of personalized prophylaxis with simoctocog alfa (Nuwiq) versus prophylaxis with EHL rFVIII products: efanesoctocog alfa 28 29 30 (ALTUVIIIO, Sanofi); turoctocog alfa pegol 31 32 (Esperoct, Novo Nordisk); efmoroctocog alfa 33 (ELOCTATE, Biogen Inc.); damoctocog alfa pegol 34 (JIVI, Bayer); and rurioctocog alfa pegol 35 (ADYNOVATE, Takeda). MAIC-based efficacy and dosing data were used to develop a cost comparison model from a United States payer perspective. For efmoroctocog alfa, damoctocog alfa pegol, and rurioctocog alfa pegol, previously published MAIC data were used. 26 New MAICs were performed to compare PK-guided, personalized prophylaxis with simoctocog alfa versus prophylaxis with efanesoctocog alfa and turoctocog alfa pegol.

## Methods

### Data Sources

IPD for simoctocog alfa were obtained from the NuPreviq study, a phase IIIb, prospective, open-label study, in male PTPs aged ≥18 years with severe hemophilia A. The study design and primary results have been published previously. 12 Key characteristics of the study are summarized in **Supplementary Appendix: Summary of prophylactic regimens of index and comparator studies** and **Supplementary Table S1** (available in the online version only). In brief, participants first underwent a PK assessment at study entry and then received a standard prophylaxis regimen (30–40 International Units [IU]/kg three times per week or every other day) for 1–3 months. Subsequently, a personalized prophylaxis regimen was established based on each participant’s PK profile, which was administered over a 6-month period. 12

Comparator data for efanesoctocog alfa and turoctocog alfa pegol were obtained from published studies. For efanesoctocog alfa, aggregate data were derived from the XTEND-1 study, a phase III, prospective, open-label trial enrolling PTPs aged ≥12 years with severe hemophilia A. 28 30 36 Participants previously on prophylaxis were assigned to once-weekly efanesoctocog alfa (50 IU/kg) for 52 weeks (Group A); while those on prior on-demand therapy received efanesoctocog alfa on-demand (50 IU/kg/dose) for 26 weeks followed by once-weekly prophylaxis (50 IU/kg) for 26 weeks (Group B). For turoctocog alfa pegol, data were sourced from the PATHFINDER-2 study, a prospective, phase III, open-label trial, in PTPs aged ≥12 years with severe hemophilia A. 32 37 38 39 Participants were treated with either prophylaxis or on-demand regimens. Those on prophylaxis received turoctocog alfa pegol (50 IU/kg) every fourth day, with twice-weekly dosing permitted at the investigator’s discretion. Further details on the XTEND-1 and PATHFINDER-2 studies are provided in **Supplementary Appendix: Summary of prophylactic regimens of index and comparator studies** and **Supplementary Table S1** (available in the online version only). Data from the on-demand treatment period (Group B, XTEND-1; PATHFINDER-2 on-demand cohort) were excluded from the MAICs.

The median duration of personalized prophylaxis in NuPreviq was 26 weeks, compared with 53 weeks (Group A) and 26 weeks (Group B prophylaxis period) in XTEND-1. 30 The mean treatment duration in PATHFINDER-2 was 42.7 weeks. 32

All data used in the MAICs were previously published and anonymized; therefore, Institutional Review Board approval and informed consent were not required.

### Feasibility Assessments

Prior to conducting each MAIC, a feasibility assessment was performed to evaluate the comparability of study characteristics, inclusion/exclusion criteria, baseline characteristics, and outcome definitions across studies.

As detailed in **Supplementary Tables S2** and **S3** (available in the online version only), the study populations across NuPreviq, XTEND-1, and PATHFINDER-2 were broadly similar, with all studies enrolling PTPs with severe hemophilia A (FVIII <1%) and at least 150 prior exposure days to FVIII products. All three studies excluded individuals with a history of FVIII alloantibodies (inhibitors) or other congenital coagulopathies. The age eligibility criterion for NuPreviq was ≥18 years, while XTEND-1 and PATHFINDER-2 included PTPs aged ≥12 years. NuPreviq and PATHFINDER-2 enrolled only males, whereas XTEND-1 permitted enrollment of males and females (one female was enrolled in Group A). PATHFINDER-2 excluded individuals with a body mass index (BMI) of >35 kg/m ^2^ .

### Statistical Methods

MAIC analyses were conducted following the methodology described by Signorovitch et al 18 40 and in accordance with best practice recommendations from the International Society for Pharmacoeconomics and Outcomes Research (ISPOR) 16 20 and the United Kingdom National Institute for Health and Care Excellence (NICE). 15

IPD for NuPreviq participants ( *N* = 65) receiving personalized prophylaxis were matched against aggregate-level data from XTEND-1 (efanesoctocog alfa Groups A [ *N* = 133] and B [ *N* = 26]), and PATHFINDER-2 (turoctocog alfa pegol, *N* = 175). Evaluated efficacy outcomes included percentage of individuals with zero bleeds (treated and untreated); percentage of individuals with zero spontaneous bleeds (treated); percentage of individuals with zero joint bleeds (treated); total annualized bleeding rate (ABR; treated and untreated); spontaneous ABR (treated); joint ABR (treated); weekly FVIII dose for prophylaxis (IU/kg); total FVIII dose required to treat bleeds (IU/kg); and percentage of bleeds treated with a single infusion.

Baseline variables selected for matching were age and body weight. Participants from NuPreviq who did not meet the key inclusion criteria for XTEND-1 or PATHFINDER-2 were excluded. Matching was performed using a method-of-moments-based logistic regression approach to estimate individual participant weights. 40 Histograms and summary statistics were generated to assess weight distributions, and effective sample size (ESS) for each comparison was calculated.

Unanchored indirect treatment comparisons were conducted using re-weighted simoctocog alfa data and comparator study data. Logistic regression models were used to estimate predicted probabilities for binary outcomes (e.g., zero bleeds). Risk differences were then calculated. Adjustment for exposure time was applied when comparing binary outcomes using a weighted logistic regression model with log-exposure time as an offset. ABRs were analyzed using weighted negative binomial regression models incorporating log-exposure time. While ABR is normalized to time, it may remain sensitive to observation period length and structure. For continuous outcomes such as FVIII dosing, mean values and mean differences were calculated using weighted or unweighted analyses, as appropriate. The 95% confidence intervals (CIs) or standard deviations (SDs) were reported for all point estimates. All analyses were performed using SAS version 9.4 and amended R code from Appendix D of NICE Technical Support document 18, 15 41 via RStudio version 4.0.3.

As an unanchored MAIC, this analysis assumes that all effect-modifying baseline characteristics are adequately accounted for by the covariates included in the weighting procedure. Matching was limited to age and body weight due to the absence of consistently reported data on other clinically relevant variables across trials. Consequently, residual confounding due to unmeasured or unreported characteristics cannot be excluded.

### Cost Analysis

Annual total costs per person were calculated using the adjusted populations from the MAICs comparing simoctocog alfa to efanesoctocog alfa (Group A) and turoctocog alfa pegol. In addition, previously published MAIC data for efmoroctocog alfa, damoctocog alfa pegol, and rurioctocog alfa pegol were used. 26 The cost model was developed from a United States payer perspective and included direct and indirect (societal) costs.

Direct costs included prophylactic FVIII therapy and treatment of breakthrough bleeds, based on average selling prices from the Medi-Span database (March 2025), assuming no wastage. 42 Direct costs also included non-drug-related health care costs of hospitalizations and emergency department visits and outpatient costs, all derived from published estimates. 43 Societal costs were calculated as 0.96 workdays lost per bleed 44 and an average weekly wage of 1,237 U.S. dollars (USD). 45 Costs are presented as annual mean costs per person, rounded to the nearest USD.

## Results

### Matching-Adjusted Indirect Comparison

#### Participant Characteristics

All 65 participants from the NuPreviq study met the eligibility criteria for inclusion in comparison with efanesoctocog alfa (XTEND-1 study). After matching to baseline characteristics (age and body weight), the ESS for NuPreviq was 35.3 for the comparison with efanesoctocog alfa Group A, and 28.4 for the comparison with efanesoctocog alfa Group B ( Table 1 ).

**Table 1:** Matching baseline characteristics for simoctocog alfa versus efanesoctocog alfa and turoctocog alfa pegol.

Characteristic	Summary statistic	Simoctocog alfa		EHL rFVIII
		Before matching	After matching	
Efanesoctocog alfa				
XTEND-1 study (Group A)				
	*N*	65	65	133
	ESS (%) ^a^	–	35.3 (54.3)	–
Age (years)	Mean (SD)	33.6 (10.0)	33.9 (15.4)	33.9 (15.3)
Body weight (kg)	Mean (SD)	80.5 (20.1)	78.0 (19.5)	78.0 (19.3)
XTEND-1 study (Group B)				
	*N*	65	65	26
	ESS (%) ^a^	–	28.4 (43.7)	–
Age (years)	Mean (SD)	33.6 (10.0)	42.8 (11.8)	42.8 (11.7)
Body weight (kg)	Mean (SD)	80.5 (20.1)	80.8 (18.1)	80.8 (18.0)
Turoctocog alfa pegol				
PATHFINDER-2				
	*N*	59 ^b^	59 ^b^	175
	ESS (%) ^a^	–	43.5 (73.8)	–
Age (years)	Mean (SD)	33.1 (10.2)	30.6 (12.6)	30.6 (12.5)
Body weight (kg)	Mean (SD)	76.1 (15.0)	75.0 (14.5)	75.0 (14.4)

Abbreviations: EHL, extended half-life; ESS, effective sample size; *N* , number of participants analyzed; rFVIII, recombinant factor VIII; SD, standard deviation.

^a^ Percentage of *N* .

^b^ Six participants excluded because the body mass index (BMI) was >35 kg/m ^2^ .

For comparison with turoctocog alfa pegol (PATHFINDER-2), six participants from NuPreviq were excluded due to a BMI of >35 kg/m ^2^ . After matching age and body weight, the ESS for simoctocog alfa was 43.5 ( Table 1 ).

#### Efficacy

**Fig. 1:**
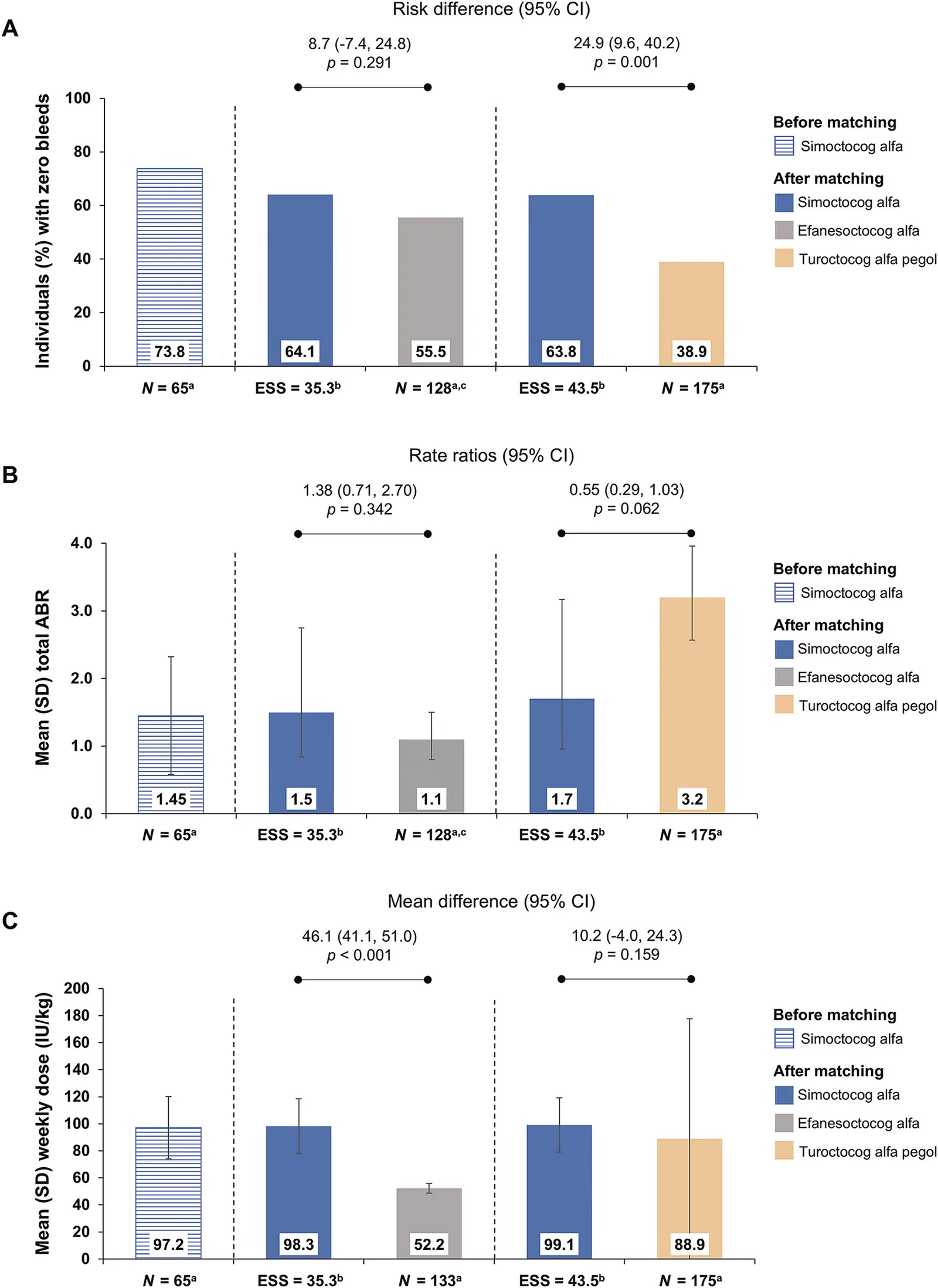
Comparison of **(A)** percentage of individuals with zero bleeds and **(B)** total ABR during personalized prophylaxis with simoctocog alfa versus efanesoctocog alfa (Group A) and turoctocog alfa pegol, and **(C)** weekly FVIII dose for prophylaxis, before and after matching. Bleeding outcomes include treated and untreated bleeds. ^a^ Published aggregate data. ^b^ Outcomes after adjustment for baseline age and body weight. ^c^ In subjects evaluable for efficacy with at least 26 weeks of exposure. ABR, annualized bleeding rate; CI, confidence interval; ESS, effective sample size; FVIII, factor VIII; N, number of individuals analyzed; SD, standard deviation.

After matching, the percentage of individuals with zero bleeds was numerically higher with simoctocog alfa compared with efanesoctocog alfa, although differences were not statistically significant: 64.1% versus 55.5% (Group A; Fig. 1A ) and 79.1% versus 73.1% (Group B; **Supplementary Table S4** [available in the online version only]). In contrast, simoctocog alfa was associated with a significantly higher proportion of individuals with zero bleeds compared with turoctocog alfa pegol (63.8% vs. 38.9%; Fig. 1A ).

Mean total ABRs were comparable between simoctocog alfa and efanesoctocog alfa Group A (1.5 [0.84, 2.75] vs. 1.1 [0.80, 1.50]; Fig. 1B ), and Group B ( **Supplementary Table S4** [available in the online version only]). The comparison between simoctocog alfa and turoctocog alfa pegol also showed no significant difference (1.7 [0.96, 3.17] vs. 3.2 [2.57, 3.96]; Fig. 1B ).

When only treated bleeds were considered, no significant differences were observed between simoctocog alfa and efanesoctocog alfa in the proportion of individuals with zero bleeds, zero spontaneous bleeds, or zero joint bleeds ( **Supplementary Tables S4 and S5** [available in the online version only]). Mean ABRs for treated bleeds were statistically higher for simoctocog alfa compared with efanesoctocog alfa Group A for total ABRs (1.5 vs. 0.7, *p* = 0.025) and spontaneous ABRs (0.9 vs. 0.3, *p* = 0.015), but not for joint ABRs (0.9 vs. 0.5, *p* = 0.161; **Supplementary Table S5** [available in the online version only]). ABRs for treated bleeds were not significantly different for simoctocog alfa versus efanesoctocog alfa Group B (1.3 vs. 0.7, *p* = 0.195; **Supplementary Table S4** [available in the online version only]). When compared with turoctocog alfa pegol, the percentage of participants with zero treated bleeds was significantly higher with simoctocog alfa (75.1% vs. 48.6%, *p* = 0.0004), while total ABRs for treated bleeds did not differ significantly (1.7 vs. 3.0, *p* = 0.086; **Supplementary Table S6** [available in the online version only]).

#### Factor VIII Dosing

Mean weekly FVIII prophylaxis dose was significantly higher with simoctocog alfa compared with efanesoctocog alfa Group A (98.3 vs. 52.2 IU/kg, *p* < 0.001) but not significantly different compared with turoctocog alfa pegol (99.1 vs. 88.9, *p* = 0.159; Fig. 1C ).

For breakthrough bleed management, the mean unadjusted total FVIII dose per bleed was not significantly different between simoctocog alfa and efanesoctocog alfa (61.3 vs. 48.2 IU/kg, *p* = 0.173) but was significantly lower than with turoctocog alfa pegol (61.3 vs. 64.6 IU/kg, *p* = 0.001; **Supplementary Fig. S1A** [available in the online version only]).

The proportion of breakthrough bleeds treated with a single infusion was significantly lower with simoctocog alfa compared with efanesoctocog alfa Group A (79.6% vs. 94.2%; *p* = 0.020), but not significantly different from turoctocog alfa pegol (79.6% vs. 77.5%, *p* = 0.857; **Supplementary Fig. S1B** [available in the online version only]). Similar results were observed in the comparison of simoctocog with efanesoctocog alfa Group B, although the difference in single infusion rates was not statistically significant ( **Supplementary Table S4** [available in the online version only]).

#### Cost Analysis

**Fig. 2:**
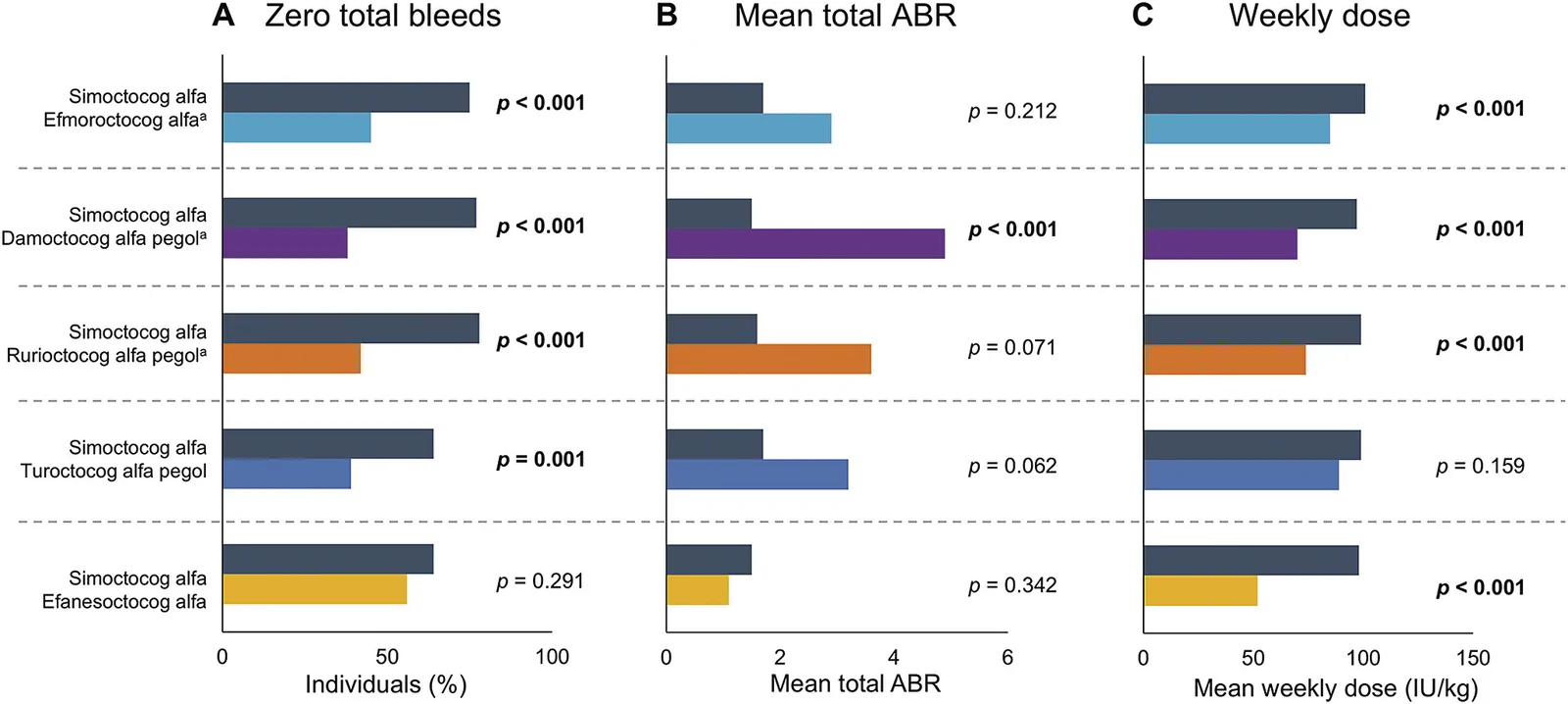
MAICs with simoctocog alfa versus EHL FVIII products. **(A)** Percentage of individuals with zero bleeds, **(B)** mean total ABR, and **(C)** mean weekly dose. ^a^ Data from published MAIC. 26 ABR, annualized bleeding rate; EHL, extended half-life; FVIII, factor VIII; IU, International Units; MAIC, matching-adjusted indirect comparison.

Inputs for the cost model were derived from the current MAICs and a previously published MAIC evaluating simoctocog alfa versus efmoroctocog alfa, damoctocog alfa pegol, and rurioctocog alfa pegol 26 ( Table 2 and Fig. 2 ).

**Table 2:** Cost analysis model input parameters.

Parameter	Simoctocog alfa	EHL rFVIII comparator				
		Efanesoctocog alfa	Turoctocog alfa pegol	Efmoroctocog alfa	Damoctocog alfa pegol	Rurioctocog alfa pegol
Participant characteristics						
Mean participant age (years)	= Comparator	33.9	30.6	29.0 26	34.6 26	31.1 26
Mean participant weight (kg)	= Comparator	78.0	75.0	71.7 26	74.2	74.1
Clinical inputs						
Total ABR (treated bleeds), simoctocog alfa versus EHL	–	1.5 versus 0.7	1.7 versus 3.0	1.7 versus 2.9 26	1.5 versus 4.9 26	1.6 versus 3.6 26
Total ABR (treated and untreated bleeds), simoctocog alfa versus EHL	–	1.5 versus 1.1	1.7 versus 3.2	1.7 versus 2.9 26	1.5 versus 4.9 26	1.6 versus 3.6 26
Dosing						
Weekly prophylaxis dose (IU/kg), simoctocog alfa versus EHL	–	98.3 versus 52.2	99.1 versus 88.9	100.7 versus 85.4 26	96.9 versus 70.1 26	99.3 versus 74.0 26
Dose to treat bleeds (IU/kg)	61.3	48.2	64.6	43.8 51	43.2	51.5 35
Direct costs (USD)						
FVIII costs per IU 42	1.24	4.64	2.20	2.34	2.45	2.10
Non-drug-related costs per person per year (USD) 43 ^,a^						
Hospitalization	6,294					
Emergency department	1,544					
Outpatient	72,077					
Indirect (societal) costs						
Days absent from work per bleed	0.96 44 ^,b^					
Median weekly U.S. salary	$1,237 45					

Abbreviations: ABR, annualized bleeding rate; EHL, extended half-life; IU, International Units; rFVIII, recombinant factor VIII; USD, United States Dollars.

^a^ Inflated to 2023 unit costs.

^b^ Ratio of workdays lost due to hemophilia to number of bleeding episodes for adults with severe hemophilia. Where no reference is provided, the data were taken from the current matching-adjusted indirect comparison analyses.

**Fig. 3:**
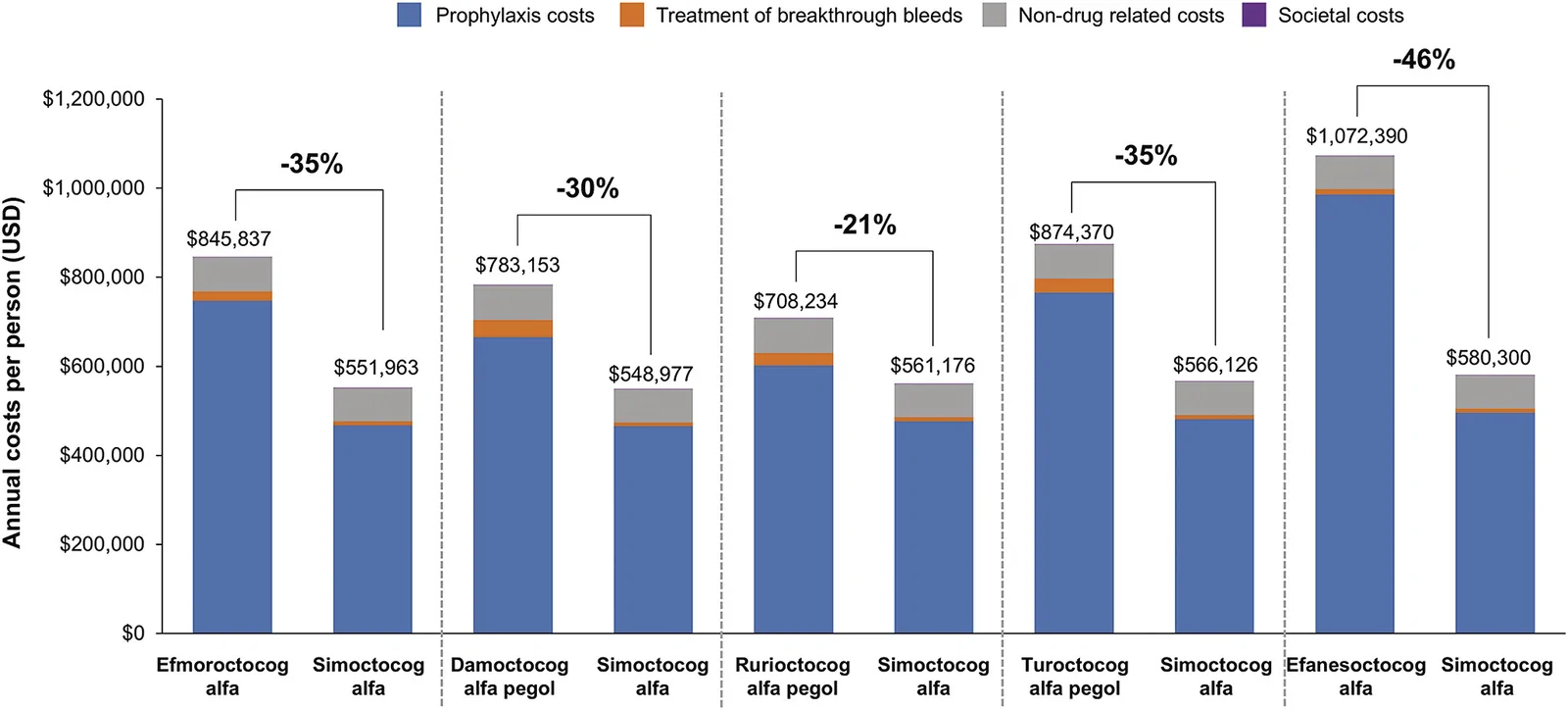
Comparison of the mean annualized cost of personalized prophylaxis with simoctocog alfa versus EHL rFVIII products. EHL, extended half-life; rFVIII, recombinant factor VIII; USD, United States Dollar.

Despite the higher weekly FVIII dose with simoctocog alfa, total annual costs per person were estimated to be $147,058 to $492,090 lower than with the EHL rFVIII comparators, representing a cost reduction of 21–46% ( Fig. 3 ). The majority of cost savings were attributable to lower drug acquisition costs with simoctocog alfa, based on 2025 average selling prices from the Medi-Span database. 42 Given the magnitude of unit price differences, cost findings are highly sensitive to pricing assumptions. Costs related to breakthrough bleeds, non-drug-related medical care, and societal productivity losses were comparable across regimens.

## Discussion

Selecting the optimal prophylactic regimen for PTPs with severe hemophilia A requires consideration not only of clinical efficacy and safety, but also relative treatment costs of available therapies. In rare diseases such as hemophilia A, direct comparisons are often unfeasible due to small sample sizes. MAIC has become an established tool to indirectly compare efficacy outcomes in such settings. 15 19 21 22 23 24 25 26 27

This MAIC analysis evaluated the relative efficacy and annual treatment costs of personalized prophylaxis with rFVIII simoctocog alfa compared with five EHL rFVIII products. Despite a higher weekly FVIII dose, simoctocog alfa was associated with lower total annual costs compared with all five EHL rFVIII products, with cost savings ranging from $147,058 to $492,090 per person. These reductions (21–46%) were primarily driven by lower drug acquisition costs, consistent with prior findings that FVIII treatment accounts for the majority of direct treatment costs in hemophilia A. 2 3

Real-world economic data further support the higher costs associated with EHL therapies. For example, an analysis of data from a U.S. commercial claims database reported total annual costs of $647,800 with SHL rFVIII and $708,928 with EHL rFVIII. 46 Another U.S. claims database analysis reported annual treatment costs of approximately $430,416 and $485,088 for the EHL rFVIIIs rurioctocog alfa pegol and efmoroctocog alfa, respectively. 47 Differences from our model likely reflect variations in age (predominantly pediatric populations), body weight, disease severity, and treatment intensity. More recently, a real-world analysis in adults with moderate or severe hemophilia A switching to efanesoctocog alfa reported a mean annual cost increase of $121,542 per person compared with prior SHL and EHL FVIII or emicizumab. 48 Additionally, a cost-effectiveness study found that the incremental cost-effectiveness ratio for efanesoctocog alfa exceeded $2 million per quality-adjusted life-year (QALY) gained versus SHL and EHL rFVIII products, which is considerably higher than conventional thresholds of $50,000 to $150,000 per QALY used in the United States, and suggested that efanesoctocog alfa would need to be priced at less than 53% of its current cost to meet conventional value thresholds. 49 These findings further emphasize the importance of comparative cost analyses grounded in adjusted efficacy data.

In our analysis, the percentage of individuals with zero bleeds and total ABRs was generally comparable between simoctocog alfa and efanesoctocog alfa. An exception was observed in the Group A comparison among patients with treated bleeds only, where efanesoctocog alfa demonstrated lower ABRs. In contrast, efficacy outcomes favored simoctocog alfa compared with turoctocog alfa pegol. These results are aligned with those from the previously published MAIC comparing simoctocog alfa to efmoroctocog alfa, damoctocog alfa pegol and rurioctocog alfa pegol, which also showed a higher percentage of individuals with zero bleeds with simoctocog alfa, albeit at a higher weekly dose. 26 Differences in follow-up duration between trials may introduce bias. Although ABR is normalized to time, it can remain sensitive to observation length, particularly in low bleeding populations, and should be considered when interpreting cross-trial comparisons.

It is important to highlight that most published MAICs in hemophilia 21 22 23 24 25 26 compare total bleeds (treated and untreated), as this aligns with primary outcomes reported in most clinical trials. Notably, the XTEND-1 study used treated bleeds as the primary endpoint. 24 However, given that even subclinical or untreated bleeds may impact joint health, 50 total ABR may represent a more meaningful endpoint in the context of prophylactic efficacy comparisons.

Although this study used MAIC-adjusted populations as inputs for both efficacy and dosing, and cost modeling followed established best practice guidance for MAIC methodology, 15 16 18 20 40 several limitations must be acknowledged. First, unanchored MAICs cannot adjust for unmeasured or unreported confounders; therefore, causal inference is not supported. Second, weighting reduced the ESS, decreasing precision of efficacy estimates and increasing uncertainty. Third, cross-trial differences introduce residual heterogeneity.

A key assumption of MAIC is that IPD are matched to the comparator study on all relevant treatment effect modifiers and outcome predictors. We acknowledge that not all baseline variables known to influence bleeding outcomes could be matched. For example, baseline joint status was not matched due to differences in data collection and definitions across studies, and bleeding history (prior ABRs) could not be matched because data were retrospective and incomplete. Differences in race, geography, prior treatment, activity level, and ABO blood group also could not be addressed owing to missing data or very small subgroup sizes. Finally, the relatively small sample sizes limited the number of baseline characteristics that could be incorporated into the matching.

This analysis is based on publicly available average selling prices and does not reflect confidential rebates or negotiated discounts. Consequently, cost relationships may differ in real-world settings. Additionally, the cost model relied on external sources for estimates of non-drug-related and societal costs, as these parameters were not captured in the clinical trials. The cost analysis was conducted from a U.S. payer perspective, and the findings may not be directly applicable to other health care systems, including Europe, where pricing mechanisms differ.

Finally, health-related quality of life (HR-QoL) outcomes were not included in the model. While HR-QoL is important, its exclusion is unlikely to have significantly altered cost estimates, given that drug costs account for the majority of total expenditures in severe hemophilia A. 2 3 However, it is accepted that a reduced dosing frequency could have a positive impact on HR-QoL.

## Conclusion

This analysis illustrates that personalized prophylaxis with simoctocog alfa is associated with substantially lower annual treatment costs across all evaluated EHL rFVIII products. Clinical outcomes were heterogeneous across comparators. While efficacy outcomes were broadly comparable in most analyses, treated total and spontaneous ABR were significantly lower with efanesoctocog alfa (Group A). Based on these MAIC and cost modeling analyses, personalized prophylaxis with simoctocog alfa may offer meaningful economic advantages while maintaining clinical outcomes across EHL rFVIII comparators. In the absence of head-to-head trials, such comparative analyses can support informed treatment decisions in clinical practice.

**What is Known about This Topic?**

- Severe hemophilia A is associated with a significant health and economic burden.
- Several recombinant factor VIII (rFVIII) products, including extended half-life (EHL) products, are now available and provide effective prevention of bleeding in persons with hemophilia A.
- Comparative evidence on the relative costs and value of prophylaxis with individual rFVIII products is limited.

**What Does This Paper Add?**

- A cost model based on a United States payer perspective) and matching-adjusted indirect comparisons was developed to evaluate the annual treatment costs of personalized prophylaxis with rFVIII simoctocog alfa compared with five EHL rFVIII products in the treatment of severe hemophilia A.
- Simoctocog alfa was associated with lower total annual treatment costs than all five EHL rFVIII products, with estimated cost savings ranging from $147,058 to $492,090 per person per year (21–46% cost reduction).

Simoctocog alfa demonstrated broadly comparable clinical outcomes across indirect comparisons while providing consistent and substantial cost savings, supporting its value as an effective, cost-efficient prophylactic option for individuals with severe hemophilia A.

## Data Availability

The datasets generated during the current study are available from the corresponding author upon reasonable request.
